# EnvIRONmental Aspects in Myelodysplastic Syndrome

**DOI:** 10.3390/ijms22105202

**Published:** 2021-05-14

**Authors:** Verena Petzer, Igor Theurl, Günter Weiss, Dominik Wolf

**Affiliations:** 1 Department of Internal Medicine V, Medical University of Innsbruck, Anichstraße 35, 6020 Innsbruck, Austria; dominik.wolf@i-med.ac.at; 2 Department of Internal Medicine II, Medical University of Innsbruck, Anichstraße 35, 6020 Innsbruck, Austria; igor.theurl@i-med.ac.at (I.T.); guenter.weiss@i-med.ac.at (G.W.)

**Keywords:** iron metabolism, myelodysplastic syndrome, bone marrow microenvironment, inflammation, oxidative stress

## Abstract

Systemic iron overload is multifactorial in patients suffering from myelodysplastic syndrome (MDS). Disease-immanent ineffective erythropoiesis together with chronic red blood cell transfusion represent the main underlying reasons. However, like the genetic heterogeneity of MDS, iron homeostasis is also diverse in different MDS subtypes and can no longer be generalized. While a certain amount of iron and reactive oxygen species (ROS) are indispensable for proper hematological output, both are harmful if present in excess. Consequently, iron overload has been increasingly recognized as an important player in MDS, which is worth paying attention to. This review focuses on iron- and ROS-mediated effects in the bone marrow niche, their implications for hematopoiesis and their yet unclear involvement in clonal evolution. Moreover, we provide recent insights into hepcidin regulation in MDS and its interaction between erythropoiesis and inflammation. Based on Tet methylcytosine dioxygenase 2 (*TET2*), representing one of the most frequently mutated genes in MDS, leading to disturbances in both iron homeostasis and hematopoiesis, we highlight that different genetic alteration may have different implications and that a comprehensive workup is needed for a complete understanding and development of future therapies.

## 1. Introduction

In contrast to many other regulatory circuits, the human body lacks a mechanism to actively get rid of excess iron. Most of the body’s daily needs for iron (~25 mg/d) are used for erythropoiesis, where iron is incorporated into the hemoglobin of newly developing red blood cells (RBC). Normally, iron absorption and iron excretion balance each other and account for approximately 2 mg/d. The remaining amount to cover the daily need for iron for erythropoiesis is established via the recycling of iron from senescent erythrocytes. This occurs in macrophages of the reticuloendothelial system, where aged erythrocytes are degraded, and the iron-containing heme is recycled [1], highlighting why disturbances in iron homeostasis often cause hematological disturbances [2,3,4]. In addition, iron’s property to switch between different oxidative states makes this micronutrient potentially toxic, as iron species possibly catalyze the Fenton reaction and generating dangerous hydroxyl radicals [5,6,7].

Thus, in simplified terms, iron overload can develop either due to increased iron uptake, increased iron supply/feed or diminished use of iron, both on a genetic and/or an interventional/pathologic basis. Iron absorption is mainly regulated via the dietary iron absorption of enterocytes in the gut. Thereby the liver-derived hormone hepcidin determines the expression of the only known iron exporter ferroportin [8,9]. The binding of hepcidin to ferroportin causes the internalization and degradation of this iron exporter and determines the rate of cellular iron uptake and export [10,11]. Diverse stimuli determine hepcidin production. However, there are several diseases where this sophisticated regulation is disrupted so that iron overload or iron deficiency develops [12,13]. There are also genetic diseases, which have uncovered the essential role of this important regulatory pathway for iron homeostasis. High iron (HFE)-associated hereditary hemochromatosis and β-thalassemia are the most prominent genetic examples [14]. In contrast to HFE-related diseases, where the genetic defect directly affects the hepcidin-ferroportin axis, the genetic defect in thalassemia causes a decreased hemoglobin production [15,16,17]. Due to this defect, erythroid maturation is hampered and typically characterized by high proliferation and a high apoptosis rate among erythroblasts, but the low output of erythroid progenitors [18,19,20]. These bone marrow characteristics have been referred to as ‘ineffective erythropoiesis’ [21]. Developing erythrocytes were found to secrete erythroferrone (ERFE), which causes hepcidin suppression in the bone marrow [22]. The low hepcidin expression enables increased iron absorption and progressive body iron accumulation with developing iron overload [23]. In addition, RBC transfusions are an integral part of therapy for anemia. Because an RBC concentrate contains ~200 mg iron, which is packed in the erythrocytes, it becomes clear that these patients are prone to develop transfusion-associated iron overload [24,25]. Of importance, as iron overload is a strong stimulus for hepcidin production, hepcidin levels are inappropriately low in patients with thalassemia, arising from the high rate of erythroid proliferation and the suppression of hepcidin formation by erythroid hormones [26,27].

Myelodysplastic syndrome (MDS) is a clonal hematopoietic stem cell disease affecting hematopoietic stem and early multipotent progenitor cells, causing dysregulated hematopoietic differentiation. MDS is characterized by peripheral blood cytopenia, especially anemia and thrombocytopenia (each ~40%), but also leukopenia (~20%), which induces fatigue, increases bleeding risk and makes affected individuals prone to infections [28,29]. Iron loading is directly linked to non-relapse mediated mortality in MDS patients after allogeneic stem cell transplantation [30]. In addition, there is a considerable risk of disease progression to acute myeloid leukemia (AML) with MDS-related features (formerly known as secondary AML: sAML), which occurs in ~30% of patients [31]. In parallel to β-thalassemia, RBC transfusions are pivotal for MDS treatment and have been shown to decrease the probability of non-leukemic causes of death, especially cardiac events [32]. However, it was found that MDS patients before transfusion dependency can already be iron overloaded, highlighting that dysregulation of iron metabolism is a disease-immanent factor [33,34]. This primary iron overload results from insufficient erythropoiesis, similar to that seen in β-thalassemia patients: high apoptosis rate despite high erythroid proliferation rate. Multiple RBC transfusions can then aggravate the iron overload, triggering secondary iron overload [35]. Another important aspect is that this secondary iron overload needs time to develop. Thus, especially low-risk (LR)-MDS patients with a higher median survival time are at higher risk for iron-related complications [36]. Correspondingly, studies have shown no difference in median survival of iron overloaded high-risk MDS patients who received iron chelation therapy than non-chelated patients [37].

In MDS, due to generating labile cellular iron, labile plasma iron and the associated production of reactive oxygen species (ROS), excess iron has been postulated to play a role in the pathogenesis, increased apoptosis rate, genomic instability, alterations in the bone marrow microenvironment, and disease progression towards AML with MDS-related features [2,38]. Multiple studies have linked transfusion dependency and concomitant iron overload with inferior outcomes [39,40,41,42]. Consequently, an underlying iron-mediated effect on disease transformation has been suggested. However, transfusion burden reflects more aggressive disease, making conclusive results difficult.

In the last decade, the understanding that iron overload is not just a bystander due to multiple RBC transfusions but also has multiple implications for the disease has steadily increased. Many reviews exist on the clinical outcome and implication of iron overload in MDS, on which we will only touch superficially in this review [2,43,44]. Herein we particularly focus on molecular aspects related to iron metabolism in MDS and discuss relevant, partly yet unanswered questions related to iron overload and MDS.

## 2. Aspects on Iron, ROS and Implications for MDS

According to the Haber–Weiss reaction, iron ions catalyze, generating hydroxyl radicals. In addition, iron is an indispensable co-factor for enzymes involved in the mitochondrial respiratory chain, where ROS are formed during oxidative phosphorylation. Excessive ROS generation is known to have multiple negative effects on cell renewal, proper differentiation and can directly cause DNA damage [6].

In MDS, elevated ROS levels were detected among all bone marrow cell types [45]. Moreover, there is evidence from the literature that higher ROS levels correlate with transfusion dependency and increased serum ferritin levels [45,46,47]. As these markers are surrogate parameters for iron overload, the overall concept of iron-induced oxidative injury has been widely accepted. This notion should be expanded by the fact that not only higher ROS production but also inadequate stress defense mechanisms contribute to the increased oxidative stress levels. Ivars et al. found higher O_2_^−^ levels together with altered expression of antioxidant systems, including catalase, glutathione and glutathione peroxidase among iron overloaded low/intermediate-1 risk MDS patients [48]. Concerning the prognostic impact of elevated iron levels in MDS patients, it was found that the presence of labile plasma iron, which is detectable once transferrin saturation exceeds 80%, is correlated to inferior overall and progression-free survival [49].

### 2.1. Effects of Iron/ROS in the Bone Marrow Niche and Hematopoietic Output

Hematopoiesis takes place in bone marrow niches, composed of different cell types (mesenchymal cells, endothelial cells, sympathetic neurons, immune cells, hematopoietic cells) and extracellular matrix, all contributing to the niche-specific microenvironment. Dysregulation of this highly complex environment is one of the hallmarks of MDS. In addition to the effects of iron and ROS on the bone matrix itself (for review, see [50]), multiple effects on the cellular composition and signaling pathways have been discovered.

In addition to the fact that ROS is deeply entangled with hematopoiesis, as a certain amount of ROS is critical for the coordinated proliferation and differentiation of hematopoietic stem cells, excessive ROS levels at first lead to higher stem cell turn-over and ultimately to stem cell exhaustion [51,52,53]. Accordingly, in vitro experiments performed with hematopoietic stem cells (HSC) from human bone marrow and in vivo studies in MDS mouse models revealed that HSC, which are exposed to increased ROS levels, have a reduced life span and are earlier exhausted, ultimately leading to lethal impairment of HSC function. One study performed in an MDS mouse model (RUNX1S291fs) specifically highlighted that aberrant frequency of HSCs is only present in mice that received regular iron injections to provoke iron overload, but not in mock-treated MDS mice [54]. In addition to the limitations of a genetic MDS mouse model and the fact that iron injections are not performed in MDS patients, these data highlight the potentially detrimental effect of iron accumulation on hematopoiesis in MDS.

Another important finding of the latter study is that the erythroid colony-forming capacity was specifically affected by iron overload, which is also relevant in humans [55,56]. In contrast, the myeloid compartment did not show such a growth restriction. The higher susceptibility to the detrimental effects of the erythroid lineage to iron overload may be explained by recent findings that intracellular oxidative stress affects erythroid development, which can be actively improved by modulation of ferroportin expression on these cells [57,58,59]. Mechanistically this effect can be traced back to the fact that higher ferroportin expression on these cells allows higher export of potentially toxic free intracellular iron. Such a mechanism could also contribute to anemia development, especially in subtypes with high hepcidin levels, although direct evidence for dysregulated ferroportin levels on erythroid precursors in patients suffering from MDS is currently lacking. If such a phenomenon turns out to be relevant, differently high levels of hepcidin between MDS subtypes further complicate such a finding (for details, see Section “Aspects on hepcidin, inflammation and MDS”). High heterogeneity in pathogenesis associated with MDS disease thus requires a detailed analysis, implying the chance to make an individual tailor-made therapy for the patient.

Multiple pathways have been found to be relevant for proper erythroid maturation (e.g., HIF-1α, NF-κB pathway) [60,61]. Moreover, iron/ROS-dependent activation of growth and differentiation factor (GDF)-11, belonging to the TGF-β superfamily, with putative GDF-11–induced over-activation of the Smad-2/3 pathway has been proposed to contribute to dyserythropoiesis in MDS [54]. GDF-11 has long been handled as the target for luspatercept, a recently approved activin receptor ligand trap for treating LR-MDS with ring sideroblasts (RS) [62]. RS are erythroid precursors with iron overloaded mitochondria, visualized via Prussian blue staining [63,64]. If the following criteria are met, diagnosing MDS-RS can be made: (1) ≥15% BM RS (≥5% in the presence of SF3B1 mutations), (2) <5% bone marrow (BM) blasts and (3) <1% peripheral blood blasts. MDS-RS have a shorter median duration of response to erythropoietin stimulating agents. Thus the diagnosis is essential, and new drugs for the amelioration of anemia are warranted [62,65,66]. While luspatercept is effective for anemia improvement, erythroid amelioration was also obvious in transgenic mice with hematopoiesis-specific GDF11 deletion [67]. Since then, Martinez et al. published that luspatercept acts via inhibition of Smad-2/3 signaling, enhancing the availability of the erythroid transcription factor GATA-1, which ameliorates dyserythropoiesis [68].

Like hematopoietic stem cells, iron overload causes pauperization of mesenchymal stromal cells (MSC) [69,70]. Via secretion of cytokines needed for self-renewal, proliferation and differentiation of HSC, MSC make an important contribution to the bone marrow microenvironment. In line with this, co-culture experiments found a functional impairment in MSC hematopoietic supportive capacity in vitro, reflected by a reduced clonal capacity of bone marrow mononuclear cells co-cultured with MSC derived from iron overloaded mice [71]. Furthermore, expression of CXCL-12, VCAM-1, IGF-1 and SCF, all growth factors critical for efficient hematopoiesis, were substantially reduced in bone marrows of iron overloaded mice [72]. Again, an abnormal ROS/HIF-1α pathway activation has been implicated in this effect [73]. Concerning the ongoing development of drugs targeting the HIF pathway, new therapeutic opportunities may open up [74]. Reduced levels of VEGF, CXCL-12 and TGF-β1 could also be observed in a study performed in iron overloaded high-risk MDS/AML patients [75]. These authors could demonstrate that the expression of p-AKT, a protein involved in cell proliferation, is significantly decreased, while the Wnt/β-catenin signal pathway has been activated in a ROS-dependent manner.

**Figure 1:**
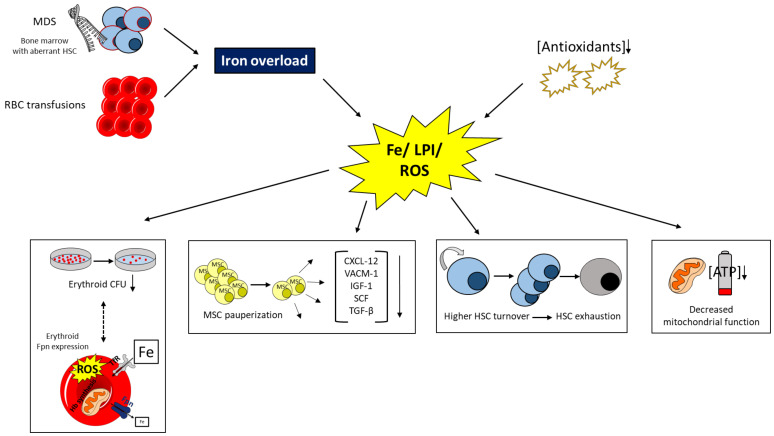
Mechanisms contributing to iron overload in patients with MDS and potential consequences. Dashed lines indicate a yet unclear interrelation. Myelodysplastic syndrome (MDS); hematopoietic stem cell (HSC); red blood cell (RBC); iron (Fe); labile plasma iron (LPI); reactive oxygen species (ROS); colony-forming unit (CFU); mesenchymal stem cell (MSC); C-X-C motif chemokine 12 (CXCL-12); vascular cell adhesion molecule 1 (VCAM-1); insulin-like growth factor 1 (IGF-1); stem cell factor (SCF); ferroportin (Fpn); adenosine triphosphate (ATP); transferrin receptor (TfR); hemoglobin (Hb).

Considering the important role of mitochondria for ROS generation, apoptosis regulation, and as sites of iron accumulation, the need to characterize mitochondrial integrity in MDS patients is obvious. In addition to the evidence of mitochondrial DNA alterations in MDS patients [76], there are reports that iron overload causes mitochondrial dysfunction in MDS patients, ultimately leading to a higher apoptosis rate [45,48,70]. While an altered energy metabolism is a hallmark of cancer, a detailed characterization of energy metabolism in MDS has just begun [77]. Interestingly, energy status was lower in MDS patients, which could be attributed to higher oxidative stress levels at the expense of decreased mitochondrial function and impaired ATP production. In addition, a higher rate of lipid peroxidation was described, which was traced back to higher malondialdehyde levels. Based on the observation that iron chelation therapy could restore the imbalance of energy metabolism and the correlation of serum ferritin with malondialdehyde levels, the authors ascribed their effects to be iron-dependent. Of note, dietary iron loading of mice is associated with impaired mitochondrial function and results in metabolic reprogramming of the Krebs cycle, glucose homeostasis and antioxidative pathways, including glutathione and cysteine formation [78,79]. Figure 1 illustrates the above-detailed effects of iron overload in MDS.

### 2.2. Effects of Iron/ROS for Genetic Instability and Leukemic Transformation

While it is known that excess iron triggers forming hydroxyl radicals, which can directly interact and harm the DNA, the role of iron for leukemic transformation is still under debate. There is literature on both that iron favors leukemogenesis [80,81,82] and that iron does not influence the transformation of MDS into AML [83,84]. However, some of these studies draw their conclusions based on the effects and outcome after therapy with iron chelation. Considering the potential effects of iron chelation besides iron-binding, these results must be interpreted with caution, and prospective trials are needed to better clarify this issue [44,85].

In addition, the fact that patients with hereditary hemochromatosis and β-thalassemia, which are also characterized by iron-induced ROS formation in the bone marrow, do not show a higher rate of MDS and AML is often stated to argue that iron does not affect transformation [86,87,88]. However, this comparison does not consider that an MDS bone marrow contains HSC and respective precursor cells with genetic lesions. Thus, from our perspective, this argument is rather weak, and further prospective studies on this topic are crucial to better understand this complex interaction. Moreover, patients with hereditary hemochromatosis have low iron content in the myeloid compartment due to increased ferroportin expression, thereby avoiding toxic iron accumulation and subsequent malignant transformation, at least within those cellular compartments [89].

Furthermore, studies linking the effect(s) of iron overload to its effects on particular genetic aberrations are still limited. One small study performed in intermediate and high-risk MDS patients found that iron overloaded MDS patients had a higher incidence of Tet methylcytosine dioxygenase 2 (*TET2*) and *ASXL1* gene mutations [75]. Both of these mutations are common in MDS patients and are associated with progression to leukemia, especially *ASXL1*, which is a well-known independent predictor of poor overall survival in these patients [90,91]. However, if there is a direct role of iron and/or ROS for generating these gene mutations is currently unclear and should be investigated in the future. In addition to genetic instability, also epigenetic abnormalities contribute to MDS disease pathogenesis, which can be induced by oxidative stress. There is evidence pointing to an effect of oxidative stress and epigenetics, but a clear concept for this interrelation in MDS is currently lacking [92,93].

**Figure 2:**
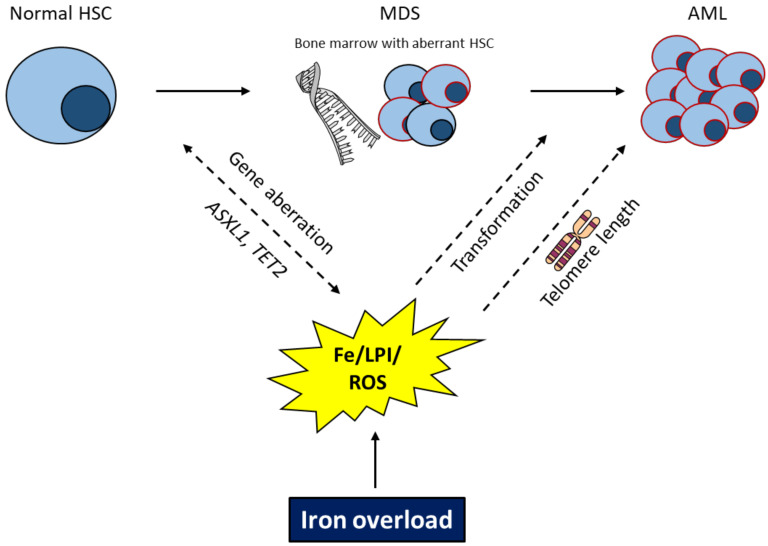
Possible roles for iron overload in MDS disease evolution. Dashed lines indicate a yet unclear interrelation. Myelodysplastic syndrome (MDS); hematopoietic stem cell (HSC); iron (Fe); reactive oxygen species (ROS); AML acute myeloid leukemia (AML).

Furthermore, iron-induced ROS is not only capable of inducing DNA damage but also causes erosion of telomeres [94]. In terms of hematopoietic cells, short telomeres predispose to chromosome instability and malignant transformation. Recently, Colla et al. found that telomere shortage interferes with the correct expression of the RNA splicing machinery in the population of common myeloid progenitors and is involved in MDS evolution [95]. Accordingly, blasts from MDS patients had shorter telomeres than controls [96,97] (Figure 2).

In contrast, independent of iron status, low/intermediate-1 risk MDS patients had higher levels of 8-Oxo-2′-deoxyguanosine levels than controls [48]. Overall, these findings suggest that MDS patients are predisposed to suffer from DNA damage. However, not only iron-induced ROS is important, but also other factors (e.g., TNF-α and hyperhomocysteinemia) that may contribute to DNA and lipid damage.

## 3. Aspects on Iron, Hepcidin, Inflammation and MDS

Hepcidin represents the master regulator of systemic iron metabolism. Hepcidin is a small liver-derived hormone, and its production is upregulated in response to high plasma iron levels, high iron stores, infection and inflammation, while anemia and hypoxia are the strongest suppressing signals. Thereby the bone morphogenetic protein (BMP)-SMAD signaling pathway, which is activated by liver endothelial cell-derived BMP6 and BMP2, is critical for hepcidin transcription. In addition, hepcidin expression can also be induced via the interleukin (IL)6-JAK-STAT and activin B-SMAD1/5/8 signaling pathway during inflammation [98,99,100,101].

Inflammation is an increasingly accepted feature of MDS [102,103,104]. Different aspects of inflammation and associated dysregulated chronic immune responses have been found to play a role (age-related inflammation, disease-associated intrinsic and extrinsic inflammation). For example, sterile inflammation via NLRP3 inflammasome activation has been linked to LR MDS. Activation of NLRP3 triggered bone marrow inflammation, expansion of myeloid-derived suppressor cells in the bone marrow, and a higher rate of hematopoietic cell damage and cell death [105,106]. Since then, it has been widely accepted that NLRP3-driven pyroptosis, a caspase-1 dependent proinflammatory form of lytic cell death, is fundamental in MDS pathology. These effects were found to be independent of genetic aberrations associated with LR-MDS. Regardless of pyroptosis in MDS, ROS has been considered as an inducer for NLRP3 inflammasome activation [107,108]. Further work has established that excessive iron is a potent inducer of the NLRP3 inflammasome via ROS-dependent mitochondrial stress or dysfunction [109]. Comparing nucleated bone marrow cells between MDS patients and healthy individuals revealed higher ROS levels in those originating from MDS bone marrows, making it feasible that inflammasome activation may originate, at least in part, from excess iron. Moreover, NLRP3 activation induces IL-1β production, which in turn stimulates hepcidin production [110]. Although direct proof of this iron-inflammasome-hepcidin axis is currently lacking, it seems feasible that a constant level of inflammation in MDS is present, culminating in a vicious circle of increased levels of hepcidin promoting cellular iron retention and ROS mediated damage.

One study performed on 113 MDS patients investigated serum hepcidin levels in more detail [111]. The authors found that hepcidin levels were overall with a wide range, but could be attributed according to the World Health Organization (WHO) classification [112]. In detail, patients with refractory anemia with ring sideroblasts (RARS) had the lowest hepcidin levels, with accordingly high values of surrogates of iron status, resulting in low hepcidin to ferritin ratio, all in all suggesting a preserved, but yet blunted regulation of iron homeostasis. In contrast, subjects with refractory anemia with excess blasts (RAEB) and chronic myelomonocytic leukemia (CMML) had the highest hepcidin levels, which were paralleled by high CRP levels rather than iron-related parameters, suggesting a higher inflammatory level and/or blast-derived cytokines that overcome hepcidin regulation by erythroid factors. Another study also identified that MDS subtypes with prominent dyserythropoiesis, such as RAEB, had significantly higher hepcidin levels [113].

**Figure 3:**
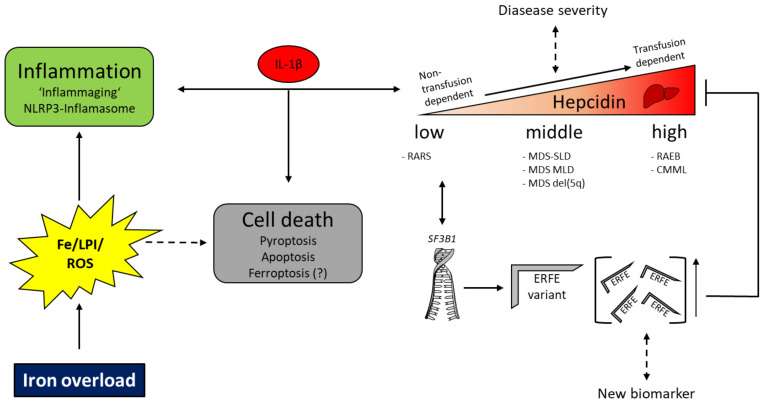
Interaction between iron, inflammation and hepcidin in patients with MDS. Dashed lines indicate a yet unclear interrelation. Myelodysplastic syndrome (MDS); hematopoietic stem cell (HSC); iron (Fe); reactive oxygen species (ROS); acute myeloid leukemia (AML); erythroferrone (ERFE); single lineage disease (SLD); multilineage disease (MLD); refractory anemia with ring sideroblasts (RARS); refractory anemia with excess blasts (RAEB); chronic myelomonocytic leukemia (CMML).

Among patients with RARS, ~90% carry a mutation in the splicing factor SF3B1. Patients carrying this mutation show inappropriately low hepcidin levels relative to their body iron stores [114]. As ineffective erythropoiesis is assumed to be present in all subtypes of MDS, it has long been speculated that additional mechanisms other than erythroid marrow activity must be present. Indeed, an ERFE variant exclusively secreted by erythroblasts from SF3B1 mutated individuals was found to be accountable for the iron phenotype in these patients. In 2014 ERFE was first described by Kautz et al., who found that ERFE is the signal derived from developing erythroblasts, which then accounts for hepcidin suppression to ensure sufficient iron availability [22]. In 2018 the finding that ERFE acts as a ligand trap, binding to BMP6 and suppressing hepcidin, has complemented this regulatory circuit [115]. In SF3B1 mutated individuals, ERFE is overexpressed (2.3-fold higher) than patients having other genetic lesions and is associated with lower hepcidin but higher ferritin levels. Of importance, to exclude an erythropoietin (EPO)-mediated effect, serum EPO levels were comparably increased in all MDS patients and increases of ERFE were independent of RBC transfusions [116]. According to one recently published study, ERFE may also serve as a prognostic marker. Despite the overall favorable prognosis of patients carrying a mutation in *SF3B1* per se, higher ERFE levels were associated with superior overall survival, independently of the international prognostic scoring system (IPSS) status. Thus, these data challenge the notion that iron overload is always a negative side effect and show that ERFE may have effects beyond hepcidin suppression, potentially serving as a new biomarker [117] (Figure 3).

Some studies also investigated the effect of RBC transfusions on hepcidin levels. Overall, hepcidin levels were higher in transfusion-dependent compared to nontransfusion dependent patients and increased over time [49,111]. Among transfusion-dependent patients, Park et al. found that high hepcidin to ferritin ratios are inversely related to many RBC transfusions and serum EPO levels, but these patients had a better and more durable response to erythropoiesis-stimulating agents [118]. This effect has been traced back to the iron content of transfusions and transient suppression of ineffective erythropoiesis associated with a hemoglobin increase.

In summary, hepcidin regulation in MDS exemplifies not only the delicate interplay of dyserythropoiesis, inflammation and iron homeostasis but also highlights that different MDS subtypes are influenced by diverse stimuli (Figure 3).

## 4. Aspects on TET2, Inflammation, Iron Metabolism and MDS

Understanding the implications of underlying mechanisms associated with certain genetic alterations opens possibilities for targeted therapies. *TET2* is frequently mutated in MDS (found in ~30%) and is well-characterized concerning the impact of this mutation on the disease [119,120].

Physiologically, Tet2 is an alpha-ketoglutarate and Fe^2+^ dependent DNA dioxygenase and is pivotally involved in epigenetic regulation via DNA demethylation. Specifically, Tet2 hydroxylates 5-methylcytosine to 5-hydroxymethylcytosine, leading to the activation of genes with cytosine-guanine-rich promoter sequences. Consequently, loss-of-function mutations of *TET2* result in DNA hypermethylation associated with dyshematopoiesis. At the stem cell level, HSC from *Tet2* knockout mice showed a higher self-renewal capacity becoming evident as an enlarged HSC compartment and HSC differentiation skewed toward monocytic/granulocytic lineages [121,122,123,124]. In line with these findings, monocytes were found to have the highest expression of this gene among all mature cell types, at least in mice [125].

In addition to those cell-intrinsic roles of Tet2 in the pathogenesis of myeloid cancers, recent investigations have also highlighted that loss of function *TET2* mutations leads to an exacerbated inflammatory phenotype. First, Tet2 is essential for resolving inflammation via Tet2-dependent recruitment of histone deacetylase 2, which deacetylates IL-6, ultimately leading to the repression of IL-6 transcription in myeloid cells [126]. There is evidence from the literature that IL-6 is overexpressed in *TET2* deficient macrophages isolated from MDS subjects [125]. Second, hyperactivation of the IL-6 pathway was also observed at the HSC level in *Tet2* knockout mice upon induction of acute inflammatory stress [127]. *Tet2* deficient hematopoietic stem and progenitor cells had a survival advantage in that they were protected from inflammation-induced damage, suggesting that increased baseline inflammation drives clonal hematopoiesis and transformation to myeloid malignancy. Third, *TET2* mutations were significantly more frequent in patients suffering from concomitant systemic autoimmune and inflammatory diseases [128]. Unsurprisingly, as cells of the adaptive immunity are also generated from HSC, there is also evidence that T cells and T cell-mediated immune responses are affected by *Tet2* deficiency [128,129,130]. MDS patients with *TET2* mutations were shown to have a better response to hypomethylating agents, such as azacytidine [131]. In summary, these findings underscore the important role of Tet2 associated with inflammation in the bone marrow microenvironment of MDS patients.

As DNA demethylation represents a crucial mechanism for erythropoiesis [132,133], an additional role for Tet2 has been established for proper erythroid development. Qu et al. found that TET2 deficiency is associated with clonal expansion of erythroid progenitor cells (CFU-E) with dysfunctional proliferation and abnormal differentiation patterns [134]. Moreover, increases in ROS production lead to alterations in DNA methylation [135,136]. High ROS levels are one hallmark of ineffective erythropoiesis but increased ROS levels are necessary for stress erythropoiesis. Experiments performed in mouse models of stress erythropoiesis revealed that ROS production induces upregulation of Tet2, which is necessary for proper differentiation [137].

Furthermore, Tet2 was found to be involved in the regulation of crucial responses for iron metabolism during stress erythropoiesis. Anemia-associated hypoxia causes EPO release, which results in erythroid proliferation and consequent suppression of hepatic hepcidin levels via ERFE and other factors, such as PDGF-BB, GDF-15 or HIF-1 [138,139,140]. Promoters of ferroportin and ERFE contain cytosine-guanine-rich promoter sequences, making them prone to demethylation processes via Tet2. Knockdown of *Tet2* led to decreased ferroportin and ERFE expression, which was paralleled by higher hepcidin levels during stress, independent of EPO levels. Collectively, the authors of this study attribute Tet2 a protective role in stress erythropoiesis via antagonization of iron-induced oxidative stress [137]. In support of this theory, we and others have shown that higher ferroportin expression on erythroid cells leads to a reduction of iron-induced stress and associated improvement of anemia in other models than MDS [57,58,59]. Moreover, *Tet2* knockdown mice develop mild anemia, show dysregulations in heme biosynthesis and iron overload at the age of 4 months, without having changes in hepcidin levels [141].

This knowledge is of importance, as TET2-inactivating mutations are also one of the most commonly mutated genes associated with a “pre-leukemic” condition, better known as clonal hematopoiesis of indeterminate potential (CHIP) [142,143,144]. Per definition, CHIP represents a clinical entity where individuals carry one or more hematological malignancy-associated mutations without other hematological malignancy defining criteria. Individuals carrying such a mutation have not only a higher risk of hematological disease and cancer but also a higher risk of cardiovascular disease [145,146]. Overall, the risk of progression to a hematological disease has been quantified with 0.5% to 1% per year [147]. While the axis between CHIP and risk of hematological disease is intuitive, the link between cardiovascular disease (including coronary heart disease, myocardial infarction with increased coronary calcification, heart failure, worse outcome after aortic valve implantation, worse cardiac remodeling) and CHIP is striking [145,146,148,149]. Regarding the pathogenesis of atherosclerosis, it was found that *Tet2* deficient macrophages may be the key cells involved in this phenomenon [146,150]. *Tet2* deficiency led to higher IL-1β levels via activation of the NLRP3 inflammasome complex. Higher IL-1β levels increased aortic expression of endothelial adhesion markers causing increased recruitment of monocytes [150]. Of interest, independent of CHIP, disturbances in iron homeostasis, as well as increased ROS levels, have been linked to the pathogenesis of atherosclerosis [151,152]. Elevated ROS lead to decreased availability of nitric oxide, being one of the most important antiatherogenic defense substances. Decreased nitric oxide facilitates leukocyte adhesion to the vessel wall and fibrous plaque formation [153]. Iron in the form of labile plasma iron has been proposed to aggravate atherosclerosis in a mouse model with impaired ferroportin function [152]. In that model, iron overload was associated with the elevated formation of proinflammatory mediators and with higher recruitment of monocytes and macrophages in atherosclerotic plaques. Thus, it is tempting to speculate that a genetic predisposition may trigger inflammatory disbalances and disturbances in iron homeostasis leading to a higher risk of cardiovascular events. However, to our knowledge, a study investigating the role of iron overload and activation of the NLRP3 inflammasome in atherosclerosis and a potential link to CHIP has yet to be performed.

## 5. Perspectives and Conclusions

**Figure 4:**
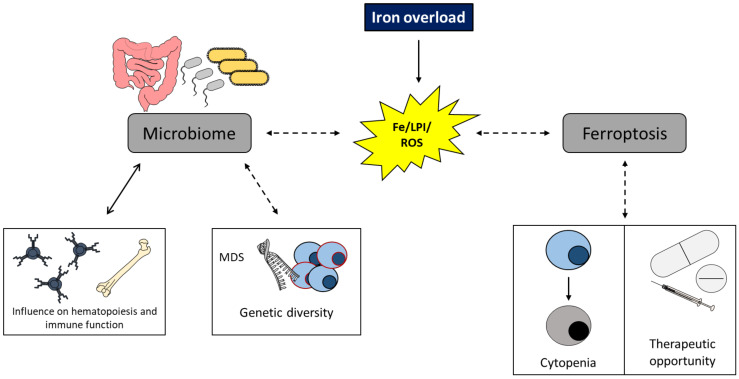
Potential consequences of iron overload in MDS on ferroptosis and the microbiome, respectively. Dashed lines indicate a yet unclear interrelation. Myelodysplastic syndrome (MDS); iron (Fe); labile plasma iron (LPI); reactive oxygen species (ROS).

As detailed above, NLRP3-driven pyroptosis contributes to MDS pathology [106]. It is to be expected that ferroptosis, a non-apoptotic form of iron-dependent oxidative cell death, will probably come into play for MDS. This form of regulated cell death is morphologically, biochemically and genetically distinct from other forms of cell death (e.g., apoptosis, pyroptosis, necroptosis and autophagy) and is triggered by iron-dependent accumulation of lipid peroxides [154,155]. While ferroptosis induction is associated with antileukemic activity in AML cell lines [43,156,157], data on MDS are currently limited. According to one recently published study, the cytosine analog used for MDS treatment, decitabine, exerts its antileukemic effect via the generation of ROS. These authors then found that the ROS-mediated effect of decitabine could be enhanced via the addition of the ferroptosis-inducing agent erastin and, vice versa, iron chelation diminished these effects [158]. Although this study hints at the involvement of ferroptosis in MDS, further studies are awaited. It also needs to be clarified whether ferroptotic cell death is intrinsically present in MDS imprinted stem cells, causing cytopenia, or if induction of ferroptosis is a novel therapeutic option (Figure 4).

Our understanding of the inflammation-iron axis will probably be further complicated once the role of the microbiome is better characterized in MDS. While perturbations of the microbiota and the axis between the gut microbiome in influencing hematopoietic immune function are increasingly recognized in diverse chronic inflammatory diseases (e.g., metabolic syndrome, inflammatory bowel disease, rheumatoid arthritis, multiple sclerosis cardiovascular diseases, graft versus host disease and cancer), there is currently no specific study concerning the microbiome composition in MDS [159]. It is possible that such studies to define patterns of alterations in the commensal microbiome associated with MDS may be impeded by MDS’s genetic diversity (Figure 4). However, according to one study performed in *Tet2*-deficient mice, disruption of the intestinal barrier with consequent translocation of gut bacteria and higher IL-6 levels triggered the establishment of preleukemic myeloproliferation [160]. In addition, it has been postulated that antibiotic treatment in mice decreases the number of HSCs and causes anemia, suggesting communication between the bone marrow and the gut microbiota [161]. Moreover, iron availability affects the composition, and metabolic activity of the microbiome and iron supplementation may specifically alter the composition of bacteria, as shown in children [162,163,164]. As most studies investigating the effect of iron overload were done in individuals or mice receiving different amounts of iron supplementation therapy, these findings cannot automatically be transferred to MDS patients, where iron overload is based on different pathophysiology. Of interest, in β-thalassemic mice, iron overload led to a gut-barrier defect, which facilitates the translocation of microorganisms and makes these mice more susceptible to sepsis [165]. However, to the best of our knowledge, there is neither direct proof that intestinal iron overload is present in MDS patients nor that iron derived from RBC transfusions and/or transfusions per se influence the microbiome. This may be subject to studies in the future. However, there is evidence that the gut microbiota can directly impact systemic iron homeostasis by regulating the expression of critical genes in iron absorption, suggesting a novel therapeutic option for iron-related disorders [166]. Das et al. recently published that intestinal iron deficiency increases the abundance of *Lactobacillus* bacteria, which directly sense intestinal iron levels and can attenuate intestinal iron absorption by the host via bacterial metabolite-mediated HIF-2α suppression [166].

Within the last two decades, the understanding of iron metabolism has steadily been expanded. Therefore, the detailed characterization of the interplay between hepcidin and its implication for regulating ferroportin expression, ERFE and erythropoiesis have substantially contributed to the iron field. In parallel, the complexity and importance of the integrity of the bone marrow niche for regulated hematopoiesis have progressively been recognized. Although iron is increasingly recognized to play a role in various aspects of MDS, multiple questions related to iron homeostasis and MDS remain to be clarified. Given that the population is aging, especially in developed countries, the incidence of MDS will rise within the next decades. Thus, a thorough assessment of interactions between iron, ROS, oxidative stress, the hematopoietic niche and hematopoietic output in MDS is needed to better understand MDS and develop new treatments.
